# Evaluation of the Biological Behavior of a Gold Nanocore-Encapsulated Human Serum Albumin Nanoparticle (Au@HSANP) in a CT-26 Tumor/Ascites Mouse Model after Intravenous/Intraperitoneal Administration

**DOI:** 10.3390/ijms20010217

**Published:** 2019-01-08

**Authors:** Chao-Cheng Chen, Jia-Je Li, Nai-Hua Guo, Deng-Yuan Chang, Chung-Yih Wang, Jenn-Tzong Chen, Wuu-Jyh Lin, Kwan-Hwa Chi, Yi-Jang Lee, Ren-Shyan Liu, Chuan-Lin Chen, Hsin-Ell Wang

**Affiliations:** 1 Department of Biomedical Imaging and Radiological Sciences, National Yang-Ming University, Taipei 112, Taiwan; jimmy10421@gmail.com (C.-C.C.); uvpxyz@gmail.com (J.-J.L.); dognew0811@gmail.com (N.-H.G.); boxes129@gmail.com (D.-Y.C.); yjlee2@ym.edu.tw (Y.-J.L.); rsliu@vghtpe.gov.tw (R.-S.L.); 2 Radiotherapy, Department of Medical Imaging, Cheng Hsin General Hospital, Taipei 112, Taiwan; chungyihwang@yahoo.com.tw; 3 Institute of Nuclear Energy Research, Taoyuan 325, Taiwan; jezon@iner.gov.tw (J.-T.C.); wjlin@iner.gov.tw (W.-J.L.); 4 Shin Kong Wu Ho-Su memorial hospital, Taipei 111, Taiwan; M006565@ms.skh.org.tw; 5 Molecular and Genetic Imaging Core/Taiwan Mouse Clinic, National Comprehensive Mouse Phenotyping and Drug Testing Center, Taipei 115, Taiwan; 6 Department of Nuclear Medicine and National PET/Cyclotron Center, Taipei Veterans General Hospital, Taipei 112, Taiwan

**Keywords:** Hybrid protein-inorganic nanoparticle, intravenous injection, intraperitoneal injection, tumor/ascites animal model

## Abstract

Colorectal cancer is one of the major causes of cancer-related death in Taiwan and worldwide. Patients with peritoneal metastasis from colorectal cancer have reduced overall survival and poor prognosis. Hybrid protein-inorganic nanoparticle systems have displayed multifunctional applications in solid cancer theranostics. In this study, a gold nanocore-encapsulated human serum albumin nanoparticle (Au@HSANP), which is a hybrid protein-inorganic nanoparticle, and its radioactive surrogate ^111^In-labeled Au@HSANP (^111^In-Au@HSANP), were developed and their biological behaviors were investigated in a tumor/ascites mouse model. ^111^In-Au@HSANP was injected either intravenously (iv) or intraperitoneally (ip) in CT-26 tumor/ascites-bearing mice. After ip injection, a remarkable and sustained radioactivity retention in the abdomen was noticed, based on microSPECT images. After iv injection, however, most of the radioactivity was accumulated in the mononuclear phagocyte system. The results of biodistribution indicated that ip administration was significantly more effective in increasing intraperitoneal concentration and tumor accumulation than iv administration. The ratios of area under the curve (AUC) of the ascites and tumors in the ip-injected group to those in the iv-injected group was 93 and 20, respectively. This study demonstrated that the ip injection route would be a better approach than iv injections for applying gold-albumin nanoparticle in peritoneal metastasis treatment.

## 1. Introduction

Colorectal cancer is the most common type of gastrointestinal cancer, accounting for over 9% of all cancer cases [1]. According to annual reports issued by the Ministry of Health and Welfare in Taiwan, colorectal cancer is second only to breast cancer in incidence and ranks third in mortality. It is also the fourth most common cause of death throughout the world [2]. Peritoneal metastasis is one of the phenomena in patients suffering from colorectal, gastric or ovarian cancer at the late phase [3,4,5,6]. Patients with peritoneal metastasis exhibit poor prognosis, with only a 12 month median survival, either with or without systemic chemotherapy [7,8].

Nanoparticles (NPs) can encapsulate poorly soluble drugs, fluorescent dyes, and imaging contrast agents and have multifunctional biological applications. In a solid tumor, the distribution of NPs is adjusted by the two main effects of passive targeting, also named enhanced permeability and retention (EPR) effect [9], and active targeting [10]. Based on the rapid development of nanotechnology, nanoparticle (NP)-based theranostic agents, also called nanotheranostic agents, have provided an opportunity to achieve individualized treatment [11].

Various sizes and shapes of gold nanoparticles (AuNPs) with different physical and chemical properties have been developed and have led to an expansion in medical applications [12,13]. For example, gold nanoclusters have been used as a fluorescent dye [14], and gold nanospheres have been employed as contrast agent in computed tomography (CT) for cancer diagnosis [15,16]. Gold nanospheres could be applied as a radiosensitizer [17]. Gold nanoshells and gold nanorods exhibit distinctive surface plasmon resonance and are widely applied in photothermal therapy [18,19,20].

Albumin, with its non-antigenicity, biocompatibility, high protein binding of various drugs (such as paclitaxel [21], doxorubicin [22], and lapatinib [23]), and biodegradability, is regarded as an ideal material in drug delivery devices [24,25]. In addition, some albumin-binding proteins, e.g., glycoprotein gp60 and secreted protein acidic and rich in cysteine, have been found to be overexpressed in breast cancer, non-small-cell lung cancer, and colon cancer [26,27]. Several albumin-conjugated drugs and albumin-based nanoparticles have been reported for cancer diagnosis and therapy [28,29,30]. In 2005, Abraxane^®^, a paclitaxel albumin-bound particle, was approved by the United States Food and Drug Administration for treatment of metastatic breast cancer, locally advanced or metastatic non-small lung cancer, and metastatic adenocarcinoma of the pancreas.

To combine the merits of both protein and inorganic nanocarriers, the development of hybrid protein-inorganic nanoparticles for drug delivery and cancer diagnostics has been progressively enlarged [30,31]. The super paramagnetic iron oxide (SPIO)-encapsulated albumin nanoparticle was developed for positron emission tomography (PET)/near-infrared fluorescence (NIRF)/magnetic resonance imaging (MRI) triple functional imaging [32] and as a drug delivery vehicle for doxorubicin [33]. Peralta and his co-workers reported that paclitaxel-loaded gold nanorod encapsulated human serum albumin nanoparticle exhibited superior tumor growth inhibition [34].

In the 1970s, intraperitoneal chemotherapy (IPC), which delivers a high concentration of cytotoxic drugs through the intraperitoneal route, was first introduced for patients with malignant ascites [35]. As the presence of a peritoneal–plasma barrier maintains a high concentration gradient of cytotoxic drugs between the peritoneal cavity and the plasma compartment, chemo drugs that are directly delivered into the peritoneal cavity show a pharmacokinetic advantage over other routes of administration. Williamson et al. reported that patients receiving a nanoparticulate formulation of paclitaxel (Nanotax^®^) through ip administration exhibited a higher and prolonged paclitaxel level in the abdominal cavity than those receiving iv injection in a phase I clinical trial [36]. Cytoreductive surgery combined IPC or hyperthermic intraperitoneal chemotherapy are two common strategies for peritoneal metastasis [37,38]. Intraperitoneal injections of Nab-paclitaxel in mice bearing OCUM-2MD3 peritoneal xenografts have shown superior therapeutic efficacy than those receiving iv injections of Nab-paclitaxel or ip injections of paclitaxel [39].

Previous studies have revealed the great potency of protein-inorganic nanoparticles for various solid tumor treatments. However, usage of these nanoparticles for treating peritoneal metastases remains unclear. This study developed a gold nanocore-encapsulated human serum albumin nanoparticle (Au@HSANP) as a drug delivery system. Indium-111 labeled Au@HSANP (^111^In-Au@HSANP) was prepared as the radioactive surrogate of Au@HSANP and used to investigate the biological behaviors in a CT-26 tumor/ascites mouse model after different routes of administration.

## 2. Results

### 2.1. Characteristics of AuNP and Au@HSANP

**Figure 1:**
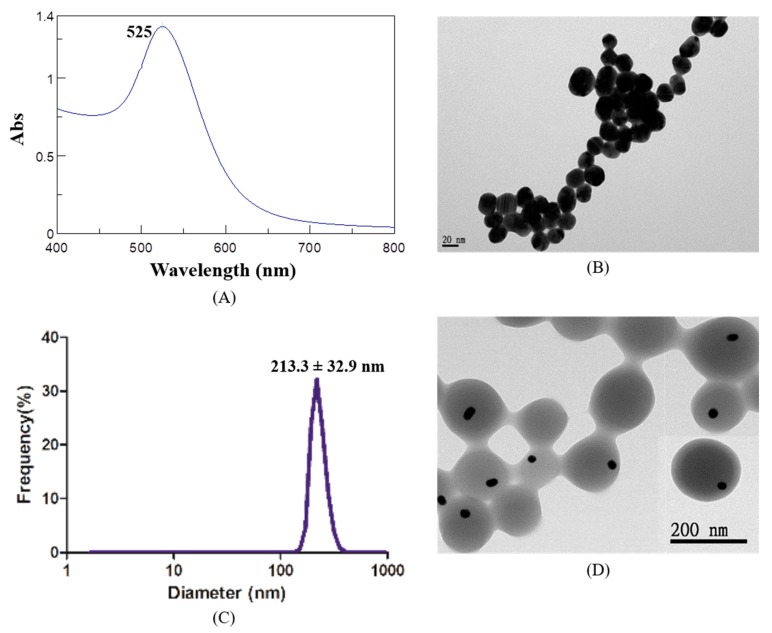

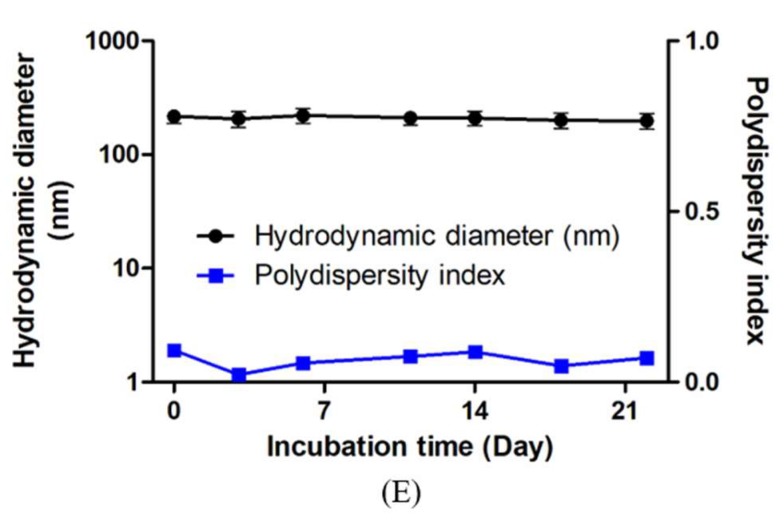
Characterizations of gold nanoparticles (AuNPs) and AuNP-encapsulated human serum albumin nanoparticles (Au@HSANPs). (**A**) The maximum absorption peak of AuNPs prepared by Turkevich’s method was at 525 nm; (**B**) the AuNPs exhibited a monodisperse structure and the average particle size was 22.5 ± 5.9 nm; (**C**,**D**) size distribution and TEM images. The hydrodynamic diameter of Au@HSANPs was 213.3 ± 32.9 nm determined by DLS; (**E**) the stability of Au@HSANPs represented by the change of particle size and PDI after incubation in deionized water at 4 °C for 22 days.

Gold nanoparticles with an average size distribution of 22.5 ± 5.9 nm (polydispersity index (PDI) = 0.16) were prepared and used for the following studies (Table 1). The ultraviolet-visible (UV/Vis) spectrum of AuNPs indicated a maximum absorption peak (λ_max_) at 525 nm, and transmission electron microscopy (TEM) images revealed the spherical shape of the AuNPs (Figure 1A,B). AuNP-encapsulated human serum albumin nanoparticles (Au@HSANPs) were prepared with an average size of 213.3 ± 32.9 nm (PDI = 0.08). Both dynamic light scattering (DLS) and TEM images illustrated a uniform distribution of Au@HSANPs (Figure 1C,D). The amount of nanoparticles in Au@HSANP solution was estimated to be 1.38 × 10^11^/mL at a concentration of 1000 ppm. The change in both particle size and PDI value of Au@HSANP was negligible after being stored in deionized water at 4 °C for more than 20 days, which indicated the high stability and undetectable aggregation of Au@HSANP (Figure 1E).

### 2.2. Cell Viability after Incubation with Au@HSANPs

**Figure 2:**
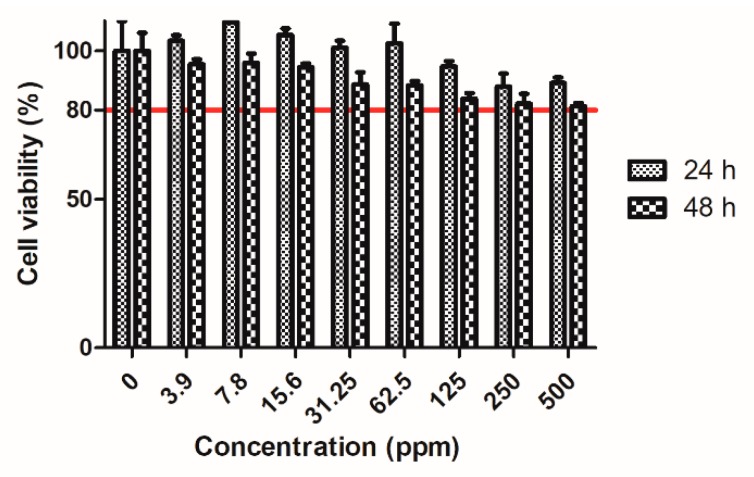
MTT assay of CT-26 cells incubated with various concentrations of Au@HSANPs. Insignificant cytotoxicity of Au@HSANPs was noticed after incubation at a concentration up to 500 ppm for 24 or 48 h. The cell viabilities of the CT-26 cells were retained >80%, as compared with the control group.

The viability of CT-26 cells after incubation with Au@HSANPs at different concentrations (0 to 500 ppm) was determined via an MTT assay, as shown in Figure 2. The high viability proves that Au@HSANP at a concentration from 0 to 62.5 ppm is not harmful to the cells after either 24 or 48 h of incubation. As the concentration up to 500 ppm, a slight reduction (around 20%) in viability was noticed. The cell viabilities of CT-26 after incubated with 500 ppm of Au@HSANP for 24 and 48 h were 89.2 ± 1.8% and 81.2 ± 1.2%, respectively.

### 2.3. Preparation and the Serum Stability of ^111^In-Labeled Au@HSANP (^111^In-Au@HSANP)

**Figure 3:**
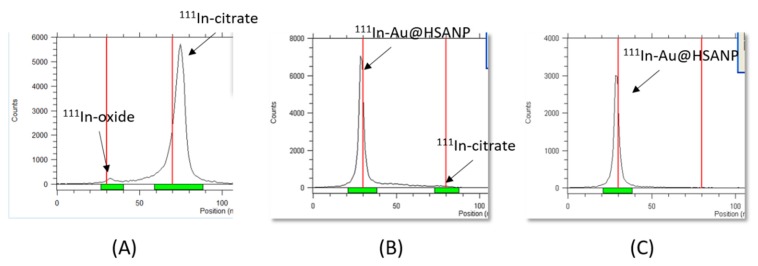
The characteristics of ^111^In-Au@HSANP. (**A**) The iTLC result of ^111^InCl_3_ using 0.5 M sodium citrate (pH 5.0) as a developing agent; (**B**) the radiolabeling efficiency of ^111^In-Au@HSANP was 95.3 ± 2.1%; (**C**) after passing through a Sepharose 4B gel column, the radiochemical purity of the purified ^111^In-Au@HSANPs was >95%.

Au@HSANP was conjugated with a metal chelator, S-2-(4-Isothiocyanatobenzyl)-diethylenetriamine pentaacetic acid (p-SCN-Bn-DTPA) at a molar ratio of 20:1 (DTPA/Au@HSANP). It was then labeled with indium-111 to afford the radioactive surrogate ^111^In-Au@HSANPs with a radiolabeling efficiency of 95.3 ± 2.1% determined by the instant thin layer chromatography (iTLC) method (Figure 3). After being purified by size exclusion chromatography, the final product was obtained with a radiochemical yield >60% and a radiochemical purity >95%. After a 72 h-incubation period in normal saline (4 °C) and fetal bovine serum (37 °C), the radiochemical activity of ^111^In-Au@HSANPs accounted for >90% in both conditions, which indicated the high in vitro stability of ^111^In-Au@HSANPs.

### 2.4. Pharmacokinetic Studies

**Figure 4:**
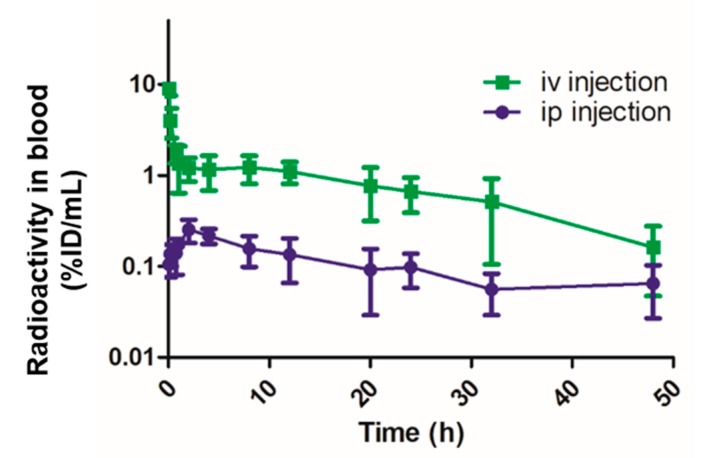
The radioactivity-time profile in blood after intravenous (iv) injection or intraperitoneal (ip) injection of ^111^In-Au@HSANPs in CT-26 tumor/ascites mice. After iv or ip injection of ^111^In-Au@HSANPs, blood from the tail vein was sampled by 1-μL capillary at designated time points and the radioactivity of each sample was counted using a gamma counter. Each value is expressed as a percentage of injected dose per milliliter (%ID/mL) and represents the mean ± SD (*n* = 5).

The dosage (10 mg/kg of body weight) of Au@HSANP used in pharmacokinetic, biodistribution studies and microSPECT imaging was based on the dosage of nab-paclitaxel, an albumin-bound 130-nm nanoparticle form of paclitaxel, for breast cancer treatment [21]. For all the in vivo studies, each mouse received 45 μCi of ^111^In-Au@HSANP (~200 μg of Au@HSANP) by either an iv or ip injection. The radioactivity-time profile in the blood after iv and ip administration of ^111^In-Au@HSANPs in the mice is displayed in Figure 4. In mice receiving iv injection of ^111^In-Au@HSANP, a biphasic profile was noticed, in which a rapid distribution phase was followed by a slow elimination phase. Low radioactivity in the blood was observed in the mice that received ip injection of ^111^In-Au@HSANPs. The pharmacokinetic parameters were calculated using WinNonlin software and are summarized in Table 2. The distribution half-life (T_1/2α_) and elimination half-life (T_1/2β_) of ^111^In-Au@HSANPs in the blood after iv injection were 0.05 and 19.7 h, respectively. The area under curve (AUC) and clearance rate (CL) were 44.5 h·[%ID/mL] and 2.2 mL/h, respectively. In mice that received ip administration of ^111^In-Au@HSANPs, the half-life in the blood calculated based on the non-compartment model was 24.4 h. The AUC and CL were 7.1 h·[%ID/mL] and 14.8 h, respectively.

### 2.5. MicroSPECT Images

**Figure 5:**
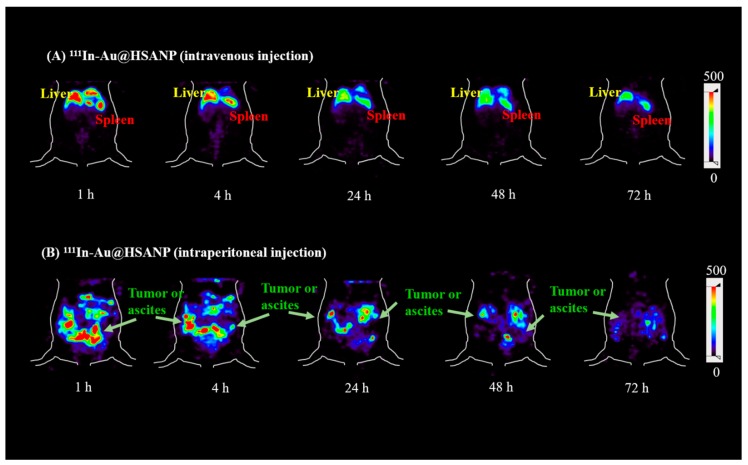
MicroSPECT images of tumor/ascites-bearing CT-26 mice after iv or ip administration of ^111^In-Au@HSANP. After receiving iv injections of ^111^In-Au@HSANP, most radioactivity accumulated in the mononuclear phagocyte system (MPS) such as the liver and spleen. After ip administration of ^111^In-Au@HSANP, remarkably less MPS accumulation was noticed. Apparent retention in the abdomen was observed until 72 h post injection.

The static microSPECT images of CT-26 tumor/ascites-bearing mice that received iv or ip injection of ^111^In-Au@HSANP are presented in Figure 5 (coronal views). After iv administration of ^111^In-Au@HSANP, a remarkable radioactivity accumulation and prolonged retention in the mononuclear phagocyte system (MPS), such as the liver and spleen, was noticed until 72 h post injection (p.i.). However, in mice that received ip injections of ^111^In-Au@HSANP, the liver and splenic uptake dramatically decreased and was almost eliminated at 24 h p.i. and was accompanied with a prolonged retention in the abdomen region.

### 2.6. Biodistribution Studies

The results of the biodistribution are summarized in Table 3 and Table 4. Among all excised organs, the spleen and liver (MPS organs) showed high radioactivity in the CT26 tumor/ascites mice at one hour post iv injection of ^111^In-Au@HSANP. The high uptake in MPS organs may account for the low radioactivity remaining in the blood (0.44 ± 0.08%ID/g at 1 h p.i.). The radioactivity accumulation in the liver and spleen slightly increased and remained high until 48 h p.i. (24.39 ± 2.03%ID/g in the liver and 45.08 ± 11.19%ID/g in the spleen), and then declined (19.06 ± 3.74%ID/g in the liver and 34.9 ± 8.5%ID/g in the spleen at 96 h p.i.). The urine radioactivity was much higher than that of the feces and indicated that the metabolites of ^111^In-Au@HSANP were mainly eliminated via renal excretion. The tumor uptake was low, at only 0.29 ± 0.10%ID/g at one-hour p.i. It rose to 0.33 ± 0.07%ID/g at 24 h p.i., and then slowly decreased to 0.21 ± 0.03%ID/g at 96 h p.i.

In mice that received ip injections of ^111^In-Au@HSANP, the uptake in the MPS organs was 0.42 ± 0.23 in the liver and 1.14 ± 0.36%ID/g in spleen at one-hour p.i was significantly lower compared to those receiving iv injections. An increasing accumulation in the liver and spleen was noticed untill 96 h p.i., indicating that ^111^In-Au@HSANP could penetrate the peritoneal capillaries into the circulation system. A remarkable tumor uptake and prolonged retention of ^111^In-Au@HSANP in the ascites after ip injections were noticed. The radioactivity retention in the ascites was 41.33 ± 12.64%ID/g at one hour p.i., which gradually decreased to 9.64 ± 3.39%ID/g at 96 h p.i.; the highest tumor uptake of 8.89 ± 2.53%ID/g was observed at 24 h p.i., declined to 1.45 ± 0.48%ID/g at 96 h p.i. The tumor uptake was 26-fold higher than that after iv injection, and the tumor-to-muscle (T/M) ratio reached 217.4 at 24 h p.i.

## 3. Discussion

Previous studies have demonstrated that opsonization followed by macrophage and Kupffer cells uptake accounts for the high MPS accumulation of most nanoparticles after iv administration [40,41,42,43]. The high level in MPS is the most significant known limitation of nanoparticulate drug delivery system. The circulation time, tumor targeting, and microdistribution in tumor regions are dependent on several factors such as the shape, size, and surface characteristics of NPs. A size less than 10 nm could avoid clearance by first pass renal filtration [44]. The blood clearance rate of particles with diameters <200 nm is slower than that for particles with diameters over 200 nm [45]. In this study, most radioactivity accumulated in MPS was noticed after iv injections of ^111^In-Au@HSANP. The results of the pharmacokinetics and biodistribution studies revealed that iv administration of Au@HSANP with an average size of 213.3 ± 32.9 nm exhibited a short distribution half-life (T_1/2α_ = 0.05 h) and a much longer elimination half-life (T_1/2β_ = 19.7 h). The radioactivity accumulation in the liver and spleen were around 16 and 40%ID/g at one hour p.i. Kinoshita et al. reported that nearly 20%ID/g of Abraxane^®^ (130 nm) accumulated in the liver at one hour post iv injection, while less than 1%ID/g accumulates in tumor lesions in CT-26 tumor-bearing nu/nu mice [46]. In 2015, Qi et al. demonstrated high liver accumulation (19.3%ID/g) of Doxorubicin-loaded HSANPs (170 nm) at one hour post iv administration [22]. Previous studies have reported that the accumulation of albumin or drugs in the lung is remarkably soon (within one hour) after iv injection of albumin [47], albumin-conjugated drugs [28], or albumin-based nanoparticles [22]. The expression of gp60 in lung microvascular endothelial cells [48] and the filtration effect of the lung capillary bed [28] may account for the noticeable lung accumulation. This study also noticed a significant radioactivity accumulation in the lung (13.31 ± 6.49%ID/g) at one hour p.i., which then declined to 1.68 ± 0.85%ID/g at 24 h p.i.

The results of the biodistribution study showed prolonged ^111^In-Au@HSANP retention in the peritoneal cavity after ip administration. The area under the radioactivity-time curves (AUCs) of the critical organs derived from the biodistribution study are summarized in Table 5. Compared with the organ AUC after iv injection, the liver and splenic AUC after ip injection were much less (about 12 and 11-fold lower). Benefitting from the low uptake in the MPS organs, the tumors and ascites in the ip injection group exhibited a 20- and 93-fold higher accumulation, respectively, than those receiving iv injections. Unlike the MPS organs, the kidneys, which are the critical excretion organ of ^111^In-Au@HSANP, displayed a similar radioactivity distribution profile in both the iv and ip-injection mice. The AUCs of kidney in iv-injection group was only 1.7-fold higher than that of ip-injection group. The lower uptake in the kidneys at one-hour post ip injection could be attributed to less ^111^In-Au@HSANP being absorbed from the peritoneum. The accumulation of ^111^In-Au@HSANP was 3.55 ± 1.00%ID/g at one-hour iv p.i., while only 0.41 ± 0.06%ID/g accumulated in the ip-injected group. These results revealed that the administration route of Au@HSANP played an important role in biodistribution but not in excretion.

The physicochemical properties of drugs, such as hydrophilicity, molecular weight, and particle size, influence, absorption in the peritoneal cavity [49,50]. Drugs with a small molecular weight exhibit a shorter residence time in the peritoneal cavity [51]. Normally, the half-life of small molecular weight drugs such as docetaxel and paclitaxel is less than 24 h after ip administration [51,52]. Drugs and NPs in the peritoneal cavity are absorbed by the capillaries and transferred to systemic circulation [53]. To lengthen the retention time of intraperitoneally administrated drugs in the peritoneal cavity, several formulations and drug delivery systems, such as microparticles, micelles and liposomes have been introduced [54]. Gelderblom et al. reported that paclitaxel entrapped in cremophor EL micelles (Taxol^®^), a nonionic castor oil derivative, exhibits a pharmacokinetic advantage for peritoneal cavity exposure after ip administration, as compared to cremophor EL-free paclitaxel formulations [55]. However, cremophor EL and ethanol-based formulations are associated with severe side effects including hypersensitivity reactions and peripheral neuropathy [56].

The size of a drug delivery system also influences the residence time in the peritoneum after ip administration. Fujiyama et al. developed a biodegradable glycolic acid–lactic acid copolymer microsphere for incorporating cisplatin (CDDP-MS) with a mean size of 19.6 μm. After ip administration, CDDP-MS exhibits a lower acute toxicity and higher retention of cisplatin in the peritoneum as compared to cisplatin solution [57]. Lu et al. reported that prolonged retention of paclitaxel-loaded microparticles (4 μm) could be noticed in the peritoneal cavity after ip administration [58]. However, Kohane et al. found several peritoneal adhesions and chronic inflammation in the peritoneum after ip injections of 5–250 μm poly(lactic-co-glycolic) acid microparticles [59]. As compared to microparticles, nanoscale particles can distribute more evenly in the peritoneal cavity and be more easily internalized by tumor cells [60,61]. Liposomes have been widely studied as potential carriers for hydrophilic and hydrophobic drugs and diagnostic agents [62]. Hirano et al. demonstrated that liposomes with a smaller size (~50 nm) pass through the lymph nodes more easily than those with a larger size (~700 nm) [63]. Studies by Dadashzadeh et al. showed that both 100 nm and 1000 nm of positively charged non-PEGylated liposomes provided greater peritoneal levels and retention. PEGylated liposome shows high peritoneal retention due to the primary tumor targeting EPR effect and also by the avoidance of macrophages present in the peritoneal cavity [64]. In our previous study, we reported that the AUCs of ascites and tumors of ^111^In-labeled PEGylated liposomal vinorelbine (IVNBPL, ~100 nm) after ip administration were 6.8- and 1.7-folds higher than that of the iv-injected group [65]. In this study, the AUC of the ascites and tumors of ^111^In-Au@HSANP in the same CT-26 tumor/ascites mouse model receiving ip administration were 93-fold and 20-fold higher than that received iv injection, respectively. In addition, it was found that the AUCs of ^111^In-Au@HSANP in the liver and spleen were 22.6 and 8.5-foldlower than that of IVNBPL, respectively. Hence, Au@HSANP could be a better drug delivery system for ip administration.

## 4. Materials and Methods

### 4.1. Materials and Reagents

Human serum albumins and glutaraldehyde solution (25% *v*/*v*) were purchased from Sigma-Aldrich Corp. (St. Louis, MO, USA). Anhydrous ethanol (>95%) was purchased from J.T. Baker Inc. (Phillipsburg, NJ, USA). Hydrogen tetrachloroaurate (III) hydrate (HAuCl_4_·3H_2_O) was purchased from Alfa Aesar (Haverhill, MA, USA). Sodium citrate dehydrate was purchased from Merck (Darmstadt, Germany). S-2-(4-Isothiocyanatobenzyl)-diethylenetriamine pentaacetic acid (p-SCN-Bn-DTPA) was purchased from Macrocyclics (Dallas, TX, USA). The ^111^In-InCl_3_ solution was obtained from the Institute of Nuclear Energy Research (Taoyuan, Taiwan). Cell culture dishes, flasks, and plastic ware were purchased from Corning Inc (Corning, NY, USA). Fetal bovine serum and cell culture medium were purchased from HyClone (Logan, UT, USA). Sepharose 4B gel and Poly-Prep chromatography columns were purchased from GE Healthcare (Chalfont St. Giles, UK) and Bio-Rad (Hercules, CA, USA), respectively.

### 4.2. Preparation of Gold Nanoparticles (AuNP)

Gold nanoparticles with a diameter of 20 nm were prepared using the Turkevich’s method [66]. All glassware and Teflon-coated magnetic bars for synthesis were cleaned using aqua regia (conc. HCl/conc. HNO_3_ = 3/1, *v*/*v*), washed with deionized water and dried prior to use. Three hundred milliliters of deionized water (dH_2_O) was heated and vigorously stirred using a Teflon-coated magnetic bar. While boiling, 3 mL of 25 mM HAuCl_4_ solution and 3 mL of 50 mM trisodium citrate were sequentially added with continuous stirring for 15 min. The obtained solution was centrifuged at 6000× *g* for 30 min. The pellets were washed in dH_2_O three times and dispersed in the dH_2_O. The UV/Vis spectrum of gold nanoparticles was recorded using a Jasco V-530 UV/VISIBLE spectrophotometer (Tokyo, Japan). The particle size and morphology were analyzed with dynamic light scattering (DLS, HORIBA, Kyoto, Japan) and transmission electron microscopy (TEM, JEOL JEM-1400plus, Tokyo, Japan), respectively. The concentration of the gold nanoparticle solution was calculated by the following formula reported by Haiss et al. [67],

$$N=\frac{A_{450}\times 10^{14}}{d^{2}\left[-0.295+1.36\exp\left(-{\left(\frac{d-96.8}{78.2}\right)}^{2}\right)\right]}$$

where *N* is number of nanoparticles in 1 mL, *A*_450_ is the absorbance at λ = 450 nm, and *d* is the particle diameter in nm.

### 4.3. Preparation of AuNP-Encapsulated Human Serum Albumin Nanoparticle (Au@HSANP)

AuNP-encapsulated human serum albumin nanoparticles were prepared using the desolvation method with a slight modification [68]. Briefly, 10 mg of human serum albumin (HSA) was dissolved in an AuNP solution (1 × 10^12^ particles/mL dH_2_O) and stirred at an ambient temperature for four hours. Next, 3 mL of ethanol was added dropwise (1 mL/min) and the mixture gradually became turbid. For crosslinking, 4.7 μL of 8% glutaraldehyde was added and then stirred at an ambient temperature for 24 h. The crude product Au@HSANP was purified through three cycles of centrifugation (3000× *g*, 10 min) and re-dispersion in dH_2_O. The final product, Au@HSANP, was kept at 4 °C before use. To investigate the stability and physiochemical properties of Au@HSANP, purified Au@HSANP was incubated in dH_2_O at 4 °C. The particle size, zeta potential, polydispersity index (PDI), and morphology of Au@HSANP were determined by DLS and TEM.

### 4.4. Cytotoxicity of Au@HSANP in CT-26 Cell Culture

An MTT assay was used for determination of the cytotoxicity of Au@HSANP in CT-26 colon adenocarcinoma cell cultures. In brief, 5000 cells were seeded in a 96-well plate for at least eight hours before the experiment. The cells were then incubated at different concentration of Au@HSANP (0–500 ppm). At 24 and 48 h post-incubation, the cells were washed twice with PBS, and then 150 μL of 0.5 mg/mL 3-(4,5-dimethylthiazol-2-yl)-2,5-diphenyltetrazolium bromide (MTT, Sigma-Aldrich Corp) was added and incubated for another four hours at 37 °C. Next, the medium was removed, and the formed formazan crystals were subsequently dissolved in 150 μL of dimethyl sulfoxide. The optical density at 570 nm of each well was recorded by using an ELISA reader (TECAN Trading AG, Mannedorf, Switzerland). The cell viability was calculated according the following formula.

$$\mathrm{Cell}\;\mathrm{viability}\;(\%)=\frac{\mathrm{Optical}\;\mathrm{density}\;\mathrm{in}\;\mathrm{group}\;\mathrm{treated}\;\mathrm{with}\;\mathrm{various}\;\mathrm{concentration}\;\mathrm{of}\;\mathrm{Au}@\mathrm{HSANP}}{\mathrm{Optical}\;\mathrm{density}\;\mathrm{in}\;\mathrm{group}\;\mathrm{without}\;\mathrm{Au}@\mathrm{HSANP}}\times 100\%$$

### 4.5. Preparation of ^111^In-Labeled Au@HSANP (^111^In-Au@HSANP)

The preparation of radioactive Au@HSANP was based on previous study with a slight modification [69]. Au@HSANP was conjugated with the bifunctional chelator, p-SCN-Bn-DTPA in a 0.05 M carbonate buffer (pH 8.5), at a molar ratio of 1:20 (HSA/DTPA) for two hours. The reaction mixture was centrifuged at 3000× *g* for 10 min and the pellet was resuspened in dH_2_O to obtain purified DTPA-Au@HSANP. The radiometal-labeling of DTPA-Au@HSANP was conducted by incubation with the appropriate amount of ^111^In-InCl_3_ in 0.1 M citrate buffer (pH 6.0) at 37 °C for 20 min. The crude solution was subjected to gel filtration chromatography with a Sepharose 4B gel column to afford the purified ^111^In-Au@HSANP with a specific activity of 10–20 mCi ^111^In/mg Au@HSANP. The labeling efficiency and radiochemical purity of ^111^In-Au@HSANP were determined using instant thin layer chromatography (iTLC) with a 0.5 M sodium citrate buffer as the developing agent.

### 4.6. Serum Stability Assay of ^111^In-Au@HSANP

The serum stability of ^111^In-Au@HSANP was assayed on the basis of a radiochemical purity determination. An adequate radioactivity of purified ^111^In-Au@HSANP was incubated in normal saline (4 °C) or fetal bovine serum (FBS, 37 °C) for four, 24, 48, and 72 h. The size exclusion chromatography with a 2 mL Sepharose 4B gel column was used to determine the radiochemical purity. At least ten fractions (0.2 mL/tube) were collected and the radioactivity of each fraction was determined. The radiochemical purity was calculated by the following formula.

$$\mathrm{Radiochemical}\;\mathrm{purity}\;(\%)=\frac{\mathrm{Sum}\;\mathrm{of}\;\mathrm{radioactivity}\;\mathrm{of}\;\mathrm{fraction}\;3–5}{\mathrm{Total}\;\mathrm{radioactivity}\;\mathrm{of}\;\mathrm{collected}\;\mathrm{fraction}}\times 100\%$$

### 4.7. Cell Culture and Tumor/Ascites Animal Model

The CT-26 colon tumor/ascites-bearing mouse model was established based on a previous study [70]. Male BALB/c mice (6–8 weeks old) were purchased from the National Laboratory Animal Center (Taipei, Taiwan). The mice were housed in cages under controlled environmental conditions, with food and water being provided ad libitum. The CT-26 ascites/tumor mice model was developed, though ip injection of CT-26 cells (2 × 10^5^) in a 0.5 mL FBS-free RPMI medium. Animal studies were conducted after 10–14 days inoculation. The animal experiment protocols were approved by the Institutional Animal Care and Use Committee of National Yang-Ming University (Taipei, Taiwan, IACUC no: 1040509r).

### 4.8. Pharmacokinetic Study of the CT-26 Tumor/Ascites-Bearing Mouse Model after iv or ip Injections of ^111^In-Au@HSANP

Ten tumor/ascites-bearing mice were randomly divided into two groups. The mice received 45 μCi of ^111^In-Au@HSANP (200 μg of Au@HSANP) by either an iv or ip injection. One microliter of blood samples was collected from the tail vein at 0.083, 0.166, 0.75, 1, 2, 4, 8, 12, 16, 20, 24, 32, and 48 h post iv/ip administration and the radioactivity of each sample was assayed using a Wallac 1470 Wizard Gamma counter (GMI, Inc., Ramsey, MN, USA). Data were expressed as the percentage of the injected dose per milliliter (%ID/mL). The pharmacokinetic parameters were calculated using WinNonlin software (version 6.1, Pharsight Corp., Mountain View, CA, USA) using a two-compartment model for the mice receiving iv injection and a non-compartment model for those receiving ip administration.

### 4.9. MicroSPECT Imaging of the CT-26 Tumor/Ascites-Bearing Mouse Model after iv or ip Injection of ^111^In-Au@HSANP

MicroSPECT images of the CT-26 tumor/ascites-bearing mice were obtained by using a microSPECT/CT scanner (FLEX Triumph Regular FLEX X-OCT, SPECT CZT 3 Head System, Gamma Medica, Northridge, CA, USA). The mice were anesthetized with 1–2% isoflurane (*w*/*v*) in 2 L of oxygen in the supine position. MicroSPECT imaging was performed at one, four, 24, 48 and 72 h after the iv/ip injection of ^111^In-Au@HSANP (340–360 μCi in 0.1 mL). Images were acquired and reconstructed using an ordered-subset expectation maximization algorithm (five iterations and eight subsets).

### 4.10. Biodistribution of CT-26 Tumor/Ascites-Bearing Mouse Model after iv or ip Injections of ^111^In-Au@HSANP

Sixteen CT-26 tumor/ascites-bearing mice were randomly divided into two groups. Each mouse received 45 μCi of ^111^In-Au@HSANP (200 μg of Au@HSANP) via either an iv or ip injection. At one, 24, 48 and 96 h post-injection, four mice in each iv-/ip-injected group were sacrificed. The tissues/organs of interest (blood, heart, lung, liver, stomach, small intestine, large intestine, spleen, pancreas, kidney, muscle, urine, feces, bladder, bone, tumor, and ascites) were harvested and weighted, and the radioactivity of each tissue/organ was counted using a gamma counter. The accumulation of ^111^In-Au@HSANP in each tissue or organ was expressed as a percentage of the injected dose per gram (%ID/g).

## 5. Conclusions

A gold nanocore-encapsulated human serum albumin nanoparticle, Au@HSANP, and its ^111^In-labeled radiosurrogate were successfully developed in this study. Both biodistribution and microSPECT imaging exhibited very significant accumulation of ^111^In-Au@HSANP in peritoneal cavity and tumor lesion after ip injection, as compared to those after iv administration. After ip injection, the area under curves (AUC) of ascites and tumor was 93 and 20-folds higher, while the organ AUC of liver and spleen was 12 and 11-fold lower, respectively, than those after iv injection. This study demonstrated that Au@HSANP, as a potential drug delivery system in treatment of peritoneal carcinomatosis, administration via ip is a better approach than via iv, and the administration route of Au@HSANP talks only in the bio-distribution, but not in excretion.

## Figures and Tables

**Table 1:** Average particle size of AuNPs and Au@HSANPs determined by dynamic light scattering (DLS).

Particle	Particle Size	Polydispersity Index (PDI)	Zeta Potential (mV)
AuNP	22.5 ± 5.9	0.16 ± 0.05	−43 ± 6
Au@HSANP	213.3 ± 32.9	0.08 ± 0.04	−46.9 ± 9

**Table 2:** The pharmacokinetic parameters of ^111^In-Au@HSANPs after intravenous (iv) or intraperitoneal (ip) injection in CT-26 tumor/ascites mice.

Parameters	Unit	iv Injection *	ip Injection **
Half-life	h	NA	24.4
T_1/2α_	h	0.05	NA
T_1/2β_	h	19.7	NA
AUC_0→∞_	h·[%ID/mL]	44.5	7.1
CL	mL/h	2.2	14.8

* two-compartment model; ** non-compartment model; NA, not applicable. T_1/2α_, distribution half-life; T_1/2β_, elimination half-life; AUC, area under the curve; and CL, clearance rate.

**Table 3:** Radioactivity distribution of ^111^In-Au@HSANP in CT-26 tumor/ascites mice at one, 24, 48, and 96 h post intravenous administration.

Organs	Mice Received iv Administration of ^111^In-Au@HSANP			
	1 h	24 h	48 h	96 h
Blood	0.44 ± 0.08	0.12 ± 0.04	0.07 ± 0.01	0.09 ± 0.03
Heart	0.17 ± 0.05	0.10 ± 0.06	0.06 ± 0.05	0.08 ± 0.03
Lung	13.31 ± 6.49	1.68 ± 0.85	0.34 ± 0.25	0.49 ± 0.40
Liver	16.09 ± 3.27	19.22 ± 2.78	24.39 ± 2.03	19.06 ± 3.74
Stomach	0.17 ± 0.06	0.09 ± 0.02	0.08 ± 0.03	0.10 ± 0.02
S.I.	0.45 ± 0.18	0.23 ± 0.10	0.15 ± 0.08	0.25 ± 0.16
L.I	0.15 ± 0.04	0.19 ± 0.11	0.14 ± 0.07	0.22 ± 0.06
Pancreas	0.15 ± 0.04	0.11 ± 0.03	0.22 ± 0.16	0.16 ± 0.02
Spleen	40.47 ± 2.36	46.45 ± 7.73	45.08 ± 11.19	34.90 ± 8.50
Kidneys	3.55 ± 1.00	5.44 ± 0.88	4.28 ± 0.67	4.36 ± 0.79
Muscle	0.03 ± 0.01	0.03 ± 0.01	0.02 ± 0.01	0.03 ± 0.01
Urine	23.68 ± 25.19	51.59 ± 18.76	22.11 ± 10.96	16.19 ± 7.41
Feces	0.03 ± 0.03	2.80 ± 1.71	1.80 ± 1.58	7.55 ± 2.38
Bladder	0.16 ± 0.06	0.22 ± 0.14	0.09 ± 0.02	0.19 ± 0.09
Bone	0.14 ± 0.05	0.29 ± 0.18	0.28 ± 0.11	0.21 ± 0.05
CT-26 tumor	0.29 ± 0.10	0.33 ± 0.07	0.19 ± 0.05	0.21 ± 0.03
Ascites	0.28 ± 0.07	0.22 ± 0.10	0.13 ± 0.02	0.22 ± 0.09
T/M ratio	9.5	11.1	9.3	7.0

Each value is expressed as a percentage of injection dose per gram of organ (%ID/g). Each value represents mean ± SD (*n* = 4). S.I., small intestine; L.I., large intestine; and T/M ratio, tumor-to-muscle ratio.

**Table 4:** Radioactivity distribution of ^111^In-Au@HSANP in CT-26 tumor/ascites mice at one, 24, 48, and 96 h post intraperitoneal administration.

Organs	Mice Received ip Administration of ^111^In-Au@HSANP			
	1 h	24 h	48 h	96 h
Blood	0.17 ± 0.07	0.15 ± 0.08	0.10 ± 0.04	0.11 ± 0.05
Heart	0.06 ± 0.03	0.08 ± 0.01	0.06 ± 0.02	0.17 ± 0.07
Lung	0.29 ± 0.18	2.78 ± 1.59	2.62 ± 0.71	15.70 ± 7.48
Liver	0.42 ± 0.23	1.11 ± 0.44	1.25 ± 0.33	3.78 ± 1.59
Stomach	0.41 ± 0.14	0.31 ± 0.10	0.33 ± 0.06	0.20 ± 0.04
S.I.	0.38 ± 0.41	0.23 ± 0.08	0.21 ± 0.09	0.22 ± 0.11
L.I	0.29 ± 0.25	0.30 ± 0.21	0.24 ± 0.04	0.19 ± 0.07
Pancreas	0.70 ± 0.19	0.55 ± 0.21	0.42 ± 0.06	0.46 ± 0.16
Spleen	1.14 ± 0.36	2.33 ± 0.18	2.38 ± 0.35	8.76 ± 3.96
Kidneys	0.41 ± 0.06	3.20 ± 0.65	3.11 ± 0.45	2.44 ± 0.35
Muscle	0.09 ± 0.12	0.04 ± 0.02	0.03 ± 0.02	0.05 ± 0.03
Urine	9.53 ± 2.36	62.81 ± 21.07	25.47 ± 19.24	10.54 ± 2.42
Feces	0.21 ± 0.24	7.76 ± 5.84	1.13 ± 0.72	3.48 ± 4.49
Bladder	0.31 ± 0.23	0.22 ± 0.07	0.31 ± 0.18	0.18 ± 0.07
Bone	0.02 ± 0.01	0.12 ± 0.07	0.06 ± 0.03	0.23 ± 0.10
CT-26 tumor	7.77 ± 2.99	8.89 ± 2.53	3.40 ± 0.94	1.45 ± 0.48
Ascites	41.33 ± 12.64	22.36 ± 5.28	12.86 ± 1.53	9.64 ± 3.39
T/M ratio	89.4	217.4	128.8	28.3

Each value is expressed as a percentage of injection dose per gram of organ (%ID/g). Each value represents mean ± SD (*n* = 4). S.I., small intestine; L.I., large intestine; and T/M ratio, tumor-to-muscle ratio.

**Table 5:** A comparison of organ area under the curve (AUC) between iv and ip injection.

Organ	iv Injection	ip Injection
Blood *	44.5	7.1
Liver	1987.9	167.3
Spleen	4057.8	365.0
Kidney	430.9	251.0
Tumor	23.1	463.3
Ascites	18.6	1736.8

AUC_0→96h_ values were derived by data of mean (h·[%ID/g]; *n* = 4 at each time point). *, AUC_0→∞_ from the results of pharmacokinetic study (unit: h·[%ID/mL]).

## Abbreviations

AUC	Area under the curve
AuNPs	Gold nanoparticle
Au@HSANP	Gold nanocore-encapsulated human serum albumin nanoparticles
CL	Clearance rate
CT	Computed tomography
EPR effect	Enhanced permeability and retention effect
HSA	Human serum albumin
HSANPs	Human serum albumin nanoparticle
ip	Intraperitoneal
IPC	Intraperitoneal chemotherapy
iv	Intravenous
MPS	mononuclear phagocyte system
NPs	Nanoparticles
PDI	Polydispersity index
p.i.	Post injection
SPECT	Single photon emission computed tomography
T/M ratio	Tumor-to-muscle ratio
TEM	Transmission electron microscopy
%ID/g	Percent of injected dose per gram
